# Characterising genome architectures using genome decomposition analysis

**DOI:** 10.1186/s12864-022-08616-3

**Published:** 2022-05-25

**Authors:** Eerik Aunin, Matthew Berriman, Adam James Reid

**Affiliations:** 1 Wellcome Sanger Institute, Cambridge, CB10 1SA UK; 2 Wellcome Centre for Integrative Parasitology, University of Glasgow, G12 8TA Glasgow, UK; 3 Wellcome/Cancer Research UK Gurdon Institute, University of Cambridge, CB2 1QN Cambridge, UK

**Keywords:** Genome architecture, Chromosome structure, Genome assembly, Parasites, Apicomplexa, Plasmodium

## Abstract

Genome architecture describes how genes and other features are arranged in genomes. These arrangements reflect the evolutionary pressures on genomes and underlie biological processes such as chromosomal segregation and the regulation of gene expression. We present a new tool called Genome Decomposition Analysis (GDA) that characterises genome architectures and acts as an accessible approach for discovering hidden features of a genome assembly. With the imminent deluge of high-quality genome assemblies from projects such as the Darwin Tree of Life and the Earth BioGenome Project, GDA has been designed to facilitate their exploration and the discovery of novel genome biology. We highlight the effectiveness of our approach in characterising the genome architectures of single-celled eukaryotic parasites from the phylum *Apicomplexa* and show that it scales well to large genomes.

**Supplementary Information:**

The online version contains supplementary material available at 10.1186/s12864-022-08616-3.

## Background

Genome architecture is the arrangement of functional elements within the genome [1] and can be thought of in a linear fashion, or in the three-dimensional arrangement found in nuclei [2]. The architecture of genomes differs greatly across the tree of life. For example, bacteria tend to have small genomes, consisting mainly of single-exon protein coding genes, often arranged in co-expressed operons, with well-defined regulatory regions [1]. Eukaryotic genomes are diverse, ranging from those that are relatively compact, with genes lacking introns (e.g. *Leishmania* spp.), to large, repeat-rich genomes, sparsely populated by multi-exon genes with large introns which employ long range regulatory interactions [3]. Although we have an excellent understanding of the evolution of protein-coding genes and how they are shaped by natural selection, we know very little of the forces that shape many aspects of genome architecture, and random drift may be the dominant force in many eukaryotic genomes [4]. Despite this, there are many features of genome architecture that are functional, and which provide clues to understanding more about the biology of an organism and its evolutionary history. For instance, in the parasitic protozoan *Plasmodium falciparum*, genes involved in evading host immunity are located in the subtelomeric regions of chromosomes where the heterochromatic environment enables clonal variability in gene expression [5, 6]. In mammals, the immunoglobulin and T-cell receptor loci comprise ordered arrays of duplicated genes, allowing the generation of variant antibody and T-cell receptor proteins [7]. Operons of co-expressed genes are found in some eukaryotes such as kinetoplastids [8] and nematodes [9]. Some fungi have genomes in which different regions have distinct evolutionary rates (https://www.sciencedirect.com/science/article/pii/S1749461320300257?via%3Dihub). There are also chromosomes that have distinct architectural patterns within a genome. These include sex chromosomes [10] and accessory B chromosomes, such as those found in plants and fungi [11]. In the nematode worm *C. elegans*, repetitive sequences have accumulated mostly at the ends of chromosomes [10]. However, some repeat families have their own distinctive patterns that are repeated across each chromosome, suggesting a variety of forces at work [12].

A key problem hampering our understanding of genome architecture has been a lack of chromosome-scale genome assemblies. However, steady advancements in the quality of long-read genome sequencing [13] and scaffolding technologies [14, 15] are beginning to solve this. Furthermore, projects such as the Darwin Tree of Life (https://www.darwintreeoflife.org/) and the Earth BioGenome Project (https://www.earthbiogenome.org/) are planning to deliver chromosome-scale assemblies for all species across the eukaryotic kingdom. A second problem is that there is no recognised approach for characterising chromosome architectures, something that would greatly facilitate studies on their evolution.

We present a new approach to characterise the linear architecture of genomes called Genome Decomposition Analysis (GDA). A genome sequence is divided into windows of arbitrary length and features are calculated for each window. Features can be derived solely from the sequence itself, including GC content, protein-coding potential, and repeat content, or include properties derived from other sources, such as sequence homology, gene expression, chromatin modifications, and recombination frequencies. The dimensionality of the resulting data matrix of windows and features is reduced and the results clustered. Parameters are explored to produce distinct clusters with a minimum of unclassified windows. Features are then identified that characterise these clusters. The pattern of clusters across chromosomes is inspected to reveal, for example, that the centres of chromosomes are distinct from the ends and that they are enriched in repeats. GDA includes an easy-to-use web application for data exploration and visualisation.

Apicomplexan parasites are well-studied due to their importance in disease and have well-understood genome architectures, making them ideal candidates for developing and testing GDA. We use GDA to: (i) refine our earlier definition of the genome architecture of the malaria parasite *P. falciparum* and characterise variation in its relatives; (ii) show that bands of repeat-rich sequence cover all chromosomes of the chicken parasite *Eimeria tenella* and compare its architecture to that of the canonical coccidian *Toxoplasma gondii*, revealing they both have distinctive but gene-poor subtelomeres; and (iii) demonstrate the potential of GDA for understanding the genome architecture of much larger genomes such as that of *Homo sapiens*.

GDA is under the MIT licence and is available from GitHub: https://github.com/eeaunin/gda

## Results

### Design of the GDA pipeline

**Fig. 1:**
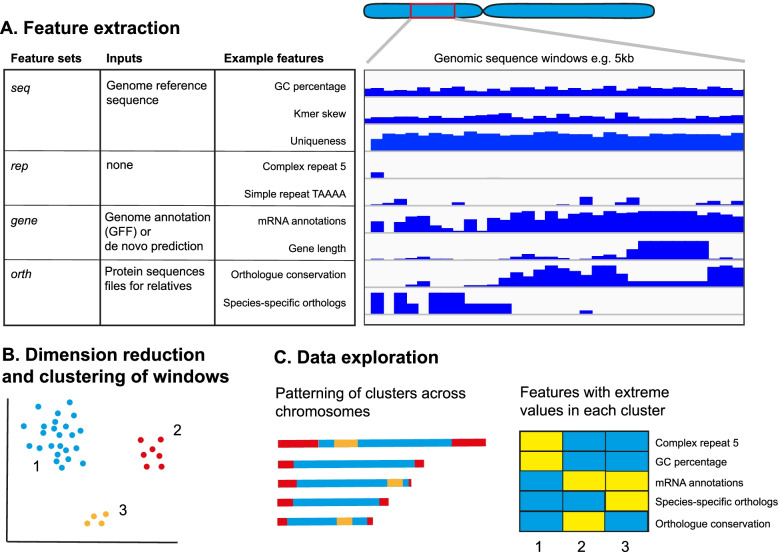
Overview of the GDA pipeline. **A** Feature sets are derived from the genome reference sequence (*seq*), repeat finding (*rep*), gene annotations (*gene*) and evolutionary relationships between genes (*orth*). The genome is divided into user-defined, non-overlapping windows (e.g. 5kbp in length) from which the value of each feature is determined. **B** The resulting matrix of feature values per window is embedded in two dimensions and clustered to identify groups of windows with similar properties. **C** The data can be explored in a number of ways using a web-browser based app. The clustering labels are mapped back to the chromosomes to highlight architectural features and a heatmap displays the features which define the clusters

We developed GDA to identify features of genome architecture from highly contiguous genome assemblies as a basis for further study of genome evolution. The tool consists of three main parts: a genomic feature extraction pipeline that calculates feature values in windows across the genome; dimension reduction and clustering of these windows; and visualisation and data exploration using a web-browser application (Fig. 1). The minimal required input for the pipeline is a genome assembly FASTA file. The features that are extracted from the FASTA file are: GC content, GC skew, AT skew, CpG dinucleotide frequency, *k-*mer frequencies, stop codon frequency, matches to a telomeric sequence motif, low complexity sequence content, tandem repeat content, coverage of simulated reads, retrotransposons, inverted repeats and repeat families (Supplementary Table 1). A more exhaustive repeat analysis can be included by running RepeatModeler which produces features describing the distribution of individual complex and simple repeats as well as features describing the sums of complex and simple repeats. Gene annotations can be used to produce bedgraph tracks of mRNA, tRNA and rRNA gene densities, average exon count, exon length and intron length. Where gene annotation files are unavailable, the pipeline can annotate genes. Likewise, if proteome FASTA files are provided for related species, the pipeline can produce bedgraph tracks based on the counts of predicted paralogs, orthologs, conserved proteins and species-specific proteins. It is also possible to add any user-generated tracks, using coordinates of the genome being analysed, to be included as input to the clustering step.

Each feature is examined in sliding windows across the genome, the output of which is stored in bedgraph files. We chose to use non-overlapping windows so that each part of the genome is classified into a distinct cluster. The size of window will strongly affect the results of the analysis. Architectural features relating to individual elements of genes such as promoters, for instance, may only be visible at higher resolution (smaller window sizes). Conversely, the presence of regions with increased numbers of intergenic repeats might only be apparent at lower resolution (larger window sizes). Larger genomes tend to have larger, more dispersed genes, so good choices of window sizes will tend to be larger in larger genomes. We try a range of window sizes to see what they tell us about a genome’s architecture. The bedgraph files can be visualised in a genome browser such as IGV [16]. The data in the bedgraph files are merged into a tab separated (TSV) file and are scaled to fit the range between 0 and 1. The resulting table is then analysed using UMAP,-a dimensionality reduction approach [17]. Dimensionality reduction algorithms aim to reduce the number of variables in a complex dataset while retaining the key properties of the data. HDBSCAN [18] is then run to detect clusters of genomic windows in the UMAP results. Next, the user can explore different values of key parameters for UMAP and HDBSCAN and compare the clusterings obtained. Low values of the *N* neighbours parameter of UMAP (n) tend to result in larger numbers of small groups of windows, while higher values tend to pull the data together into smaller numbers of large groups. The *minimum cluster size* parameter of HDBSCAN (c) limits the size of clusters identified in the UMAP results. It is not straightforward to determine what are the best parameters to use for the clustering and so users can assess clusterings generated with a range of parameters. The coherence of the clustering can be measured with the silhouette score – a high score, closer to one, means that windows are well clustered. However, we also consider the proportion of windows which fall outside of clusters, which we would like to be low, and the total number of clusters. Very low or very high numbers of clusters tend to be less informative about genome architecture. When suitable parameter values have been chosen, the clustering and analysis script is run, producing a set of output files. One of the output files is a BED file that marks which cluster each genomic window belongs to. We identify characteristic features for each cluster using the two-sample Kolmogorov-Smirnov test. Using this test, we compare the distribution of values for a feature in a cluster against the distribution of values for that feature in all other clusters. The test is two-sided and we look to see whether the test statistic *D*, is significantly greater (*D* +) or lower (*D-*) than expected. Where *D* + is significant, the cluster being examined tends to have higher values for that feature, while they tend to be lower if *D-* is significant. In some cases, both *D* + and *D-* are significant, indicating the distribution of values for that feature in the cluster of interest is more spread out than for other clusters. The clustering and analysis results can be explored using the GDA web app that includes a scatter plot of clustered windows, how these clusters are arranged over the genome, heatmaps of features enriched in each cluster, and the cluster composition of each chromosome.

### Redefining *Plasmodium falciparum* genome architecture

A complete chromosomal genome assembly of the human malaria parasite *Plasmodium falciparum* has been available for almost 20 years [19, 20]. Given the importance of the *P. falciparum* genome as a reference for studying one of the most persistent and deadly human infectious diseases, it is not surprising that there is a good understanding of its architecture. Each chromosome end has a region adjacent to the telomere known as the subtelomere. In *Plasmodium* spp. subtelomeres contain multiple members of expanded gene families which are highly variable between species. The central, core regions of chromosomes contain much more well conserved genes.

**Fig. 2:**
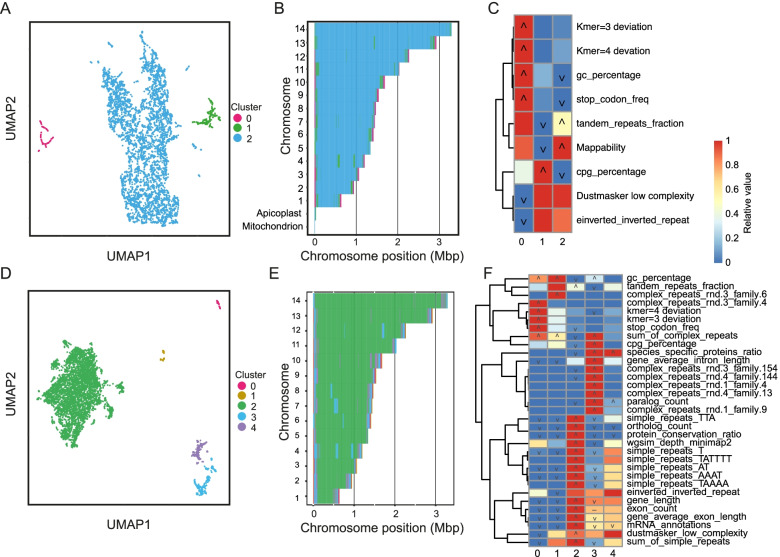
GDA analysis of the *Plasmodium falciparum* genome. **A** UMAP embedding (*n* = 5) and HDBSCAN2 clustering (c = 50) of 5kbp windows using simple features derived from the genome sequence (*seq* feature set). **B** Projection of clusters onto the chromosomes highlights the localisation of cluster 0 windows at the very ends of chromosomes, with cluster 1 windows adjacent to these and within the cores of some chromosomes. **C** Heatmap showing features enriched in each cluster with *seq* feature set. Colours indicate the relative value of the feature in each cluster (red = highest, blue lowest), icons indicate significance (‘∧’ = KS test greater p-value <  = 1e-20, ‘∨’ = KS test lesser p-value <  = 1e-20, ‘-’ = great and lesser *p*-values <  = 1e-20) (D) UMAP embedding (*n* = 20) and HDBSCAN2 clustering (c = 50) of 5kbp windows with *seq* + *gene* + *rep* + *orth* feature set. **E** Projection of clusters onto chromosomes shows that the additional features break the subtelomeric regions into four distinct regions and that two types of islands (clusters 3 and 4) interrupt the core (cluster 2) on some chromosomes. **F** Heatmap showing features enriched in each cluster with all features

We first tested the ability of GDA to identify the known architectural features of the *P. falciparum* genome using only features derived from the genome sequence itself (*seq* feature set). We chose a window size of 5kbp to capture a small number of genes per window and to reflect the resolution of the genome architecture we expect to see. For genomes where the architecture is unknown we recommend choosing several window sizes and comparing results. We explored a range of UMAP *nearest neighbour* (n) and HDBSCAN *minimum cluster size* (c) parameters but picked *n* = 5 and c = 50 as these resulted in a relatively high silhouette score of 0.28, with 100% of windows being classified (Sup Fig. 1; Fig. 2A). The three resulting clusters defined the core (cluster 2), the multigene family arrays (cluster 1) and the GC-rich Telomere Associated Repeat Element (TARE) region adjacent to the telomeres (cluster 0; Fig. 2B). The core was characterised by uniqueness of sequence (simulated mapping coverage of 9.94x, *p* = 1.23e-96), tandem repeats (*p* = 1.09e-36) and low GC percentage (18.6% in the core vs. 22.2% across other clusters, *p* = 8.43e-43) (Fig. 2C). The multigene family-rich regions were defined by high CpG percentage (0.96 vs. 0.66, *p* = 9.00e-32) and low uniqueness as measured by mapping coverage of simulated reads (3.9 × compared to the maximum 10 × generated by the algorithm, *p* = 4.44e-131). This was caused by highly similar regions in tandemly duplicated gene clusters. The TARE region was defined by high GC percentage (32.4%, KS test *p*-value = 4.90e-146), high stop codon frequency (0.24, KS test p-value 1.43e-87), and *k*-mer deviation (3-mer, *p* = 1.30e-69 and 4-mer, *p* = 6.69e-48) (Fig. 2C). This definition of *P. falciparum* genome architecture required only the genome sequence and simple parameters derived from it yet characterised both the relatively GC-rich telomere-adjacent regions, gene-family rich subtelomeres and the conserved core.

**Fig. 3:**
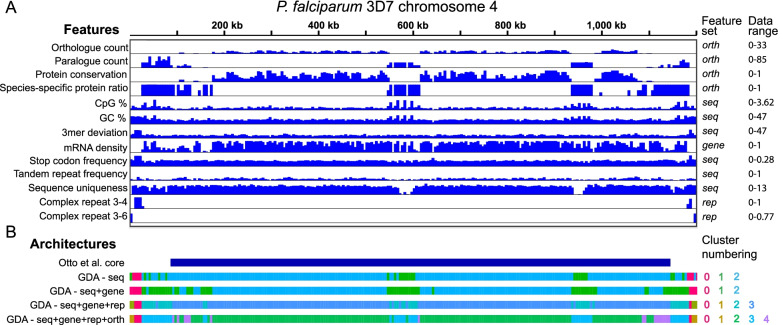
Detailed view of *Plasmodium falciparum* chromosome 4. **A** A selection of the features used as input to GDA displayed across the 1.2Mbp chromosome 4. These features were identified as significant in one or more clusters of one or more GDA runs. Data range indicates minimum and maximum values for the y axis of each feature. **B** Chromosome architectures generated using different feature sets with comparison to the definition of Otto et al*.* which captures only the core [21]. GDA was run with basic sequence features, with the addition of gene annotation, with gene annotations and complex repeat finding, with gene annotations, complex repeat finding and orthology analysis

To improve on this definition of the genome architecture we generated features from three additional sources, adding protein-coding gene annotations (*seq* + *gene*), then repeat classification (*seq* + *gene* + *rep*) and finally protein-coding gene conservation (*seq* + *gene* + *rep* + *orth*). The *gene* feature set adds an additional eight features. The *rep* feature set added 35 complex repeat features, 85 simple repeat features and features for the sum of all complex and the sum of all simple repeats. The number of repeat features will vary between genomes depending on the complexity of repeats. The *orth* feature set added four features describing the homologous relationships between *P. falciparum* protein-coding genes and those in a selection of related species (Supplementary Table 1). For each of these feature sets we re-ran the feature extraction pipeline and chose clustering parameters that minimised the number of unclustered windows, while providing several large, well-separated clusters, with a high silhouette score. Adding gene annotations altered the definition of the subtelomeres, extending them inwards towards the centromeres. This was because the extended regions are similarly less gene-dense compared to the core regions of the chromosomes (Fig. 3). Adding repeat classification (*seq* + *gene* + *rep*) differentiated the TARE2-5/SB-2 region (named *complex_repeats_rnd-3_family-6* by GDA) closest to the telomeres [19] from the TARE6/SB-3/rep20 repeat (named *complex_repeats_rnd-3_family-4* by GDA). Repeat identification altered the definition of the multigene family regions to be more like that found when only sequence-based information was used. This was because the larger multigene families were identified as repeats and this excluded the smaller multigene families. Including all this information, plus analysis of gene conservation (*seq* + *gene* + *rep* + *orth*) allowed improved definition of the multigene family-containing subtelomeric cluster-all 65 *var* genes, 155/157 *rifin* genes and 31/32 *stevor* genes overlapped cluster 3. It also highlighted the more conserved, distal subtelomeric regions containing smaller gene families, where there is conservation of synteny within *P. falciparum*, but not between species (cluster 4; Fig. 2D-F; Fig. 3B). Our analysis provides a much richer definition of the genome architecture compared to that developed previously [21]. One reason for large gene families such as *var* and *rif* being localised to the ends of chromosomes appears to be that they are regulated by facultative heterochromatin formation in the subtelomeres (typified by the binding of Heterochromatin Protein 1 – HP1) and this method of gene expression control extends to internal *var* gene arrays [6]. The role of HP1 in regulating other gene families is less well studied. We examined HP1 occupancy measured by ChIP-seq across GDA clusters, using an existing dataset [6]. We found that HP1 occupancy was higher in the var/rif-containing cluster 3 regions than cluster 4 regions, which contain the more well-conserved, smaller multigene families, as expected (Sup Fig. 2). However, while internal and subtelomeric cluster 3 regions had similar HP1 occupancy, genes in internal cluster 4 regions had less HP1 bound than those in subtelomeric cluster 4 regions. This suggests that whereas *var*-gene-containing cluster 3 regions are regulated by HP1 to a similar extent in subtelomeric and internals locations, the multigene families in cluster 4 are less strongly regulated by HP1 in internal locations.

### Defining the unique arrangement of the *P. knowlesi* genome

**Fig. 4:**
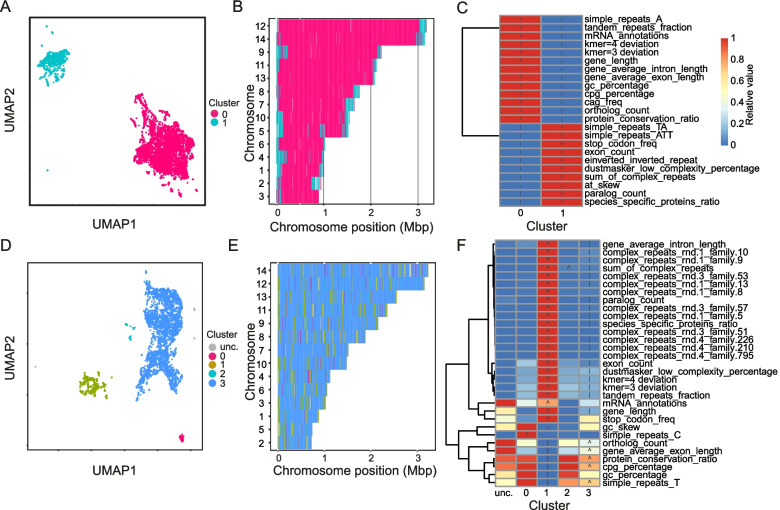
GDA analysis of the *Plasmodium vivax* P01 and *P. knowlesi* H genomes. **A** The *P. vivax* genome neatly separates into two clusters with *seq* + *rep* + *gene* + *orth* feature sets. **B** These represent core (magenta) and subtelomeric (cyan) regions. **C** The clusters are typified, amongst other things, by having one-to-one orthologous genes versus highly paralogous species-specific genes, respectively. In the heatmap colours indicate the relative value of the feature in each cluster (red = highest, blue lowest), icons indicate significance (‘∧’ = KS test greater p-value <  = 1e-20, ‘∨’ = KS test lesser p-value <  = 1e-20, ‘-’ = great and lesser p-values <  = 1e-20). **D** *P. knowlesi* separated into four clusters. **E** None of the clusters were localised to the subtelomeres. **F** The cluster with large species-specific gene families equivalent to the subtelomeric cluster of *P. vivax* (cluster 1; green) is dispersed throughout each chromosome

Most *Plasmodium* species have similar genome architectures to *P. falciparum*, with large multigene families localised largely to the subtelomeres. The clear exception is *P. knowlesi*, a related species that also causes malaria in humans and other primates. In this species, the largest, most rapidly evolving multigene families (in this case *sicavar* and *pir*) are found in islands throughout chromosomes associated with telomere-like repeats [22]. We used this example to examine the utility of GDA for comparative genomics—identifying differences in architecture between related species. *P. vivax* is a closer relative to *P. knowlesi* than *P. falciparum* but has a genome split into gene-family rich subtelomeric regions and a well conserved core like *P. falciparum*. We ran GDA on the *P. vivax* genome with a *seq* + *gene* + *rep* + *orth* feature set, identifying two clusters characterising the whole genome. This confirmed that like *P. falciparum*, most *P. vivax* chromosomes are made up of cores with well-conserved genes (cluster 0; Fig. 4A-C), while the subtelomeres contain species-specific genes with high numbers of paralogues (cluster 1).

GDA analysis of *P. knowlesi* resulted in four clusters with 82.96% of the windows assigned falling into cluster 3, representing well-conserved genes (Fig. 4D-F). Note that while the window size was the same, clustering parameters were slightly different to those used for *P.vivax*. Clustering parameters were chosen to separately to maximise the silhouette score while minimising the number of unclustered windows in each case. This may mean that some detailed comparisons of the datasets are not appropriate. Cluster 1 (12.41%) represented the multi-gene family-rich regions which are interspersed throughout the chromosomes, rather than concentrated towards the telomeres as observed in other *Plasmodium* spp. This cluster was also enriched for complex repeat families (*sum of complex repeats* *p* = 0). Several of these repeat families contained telomere-like repeats (e.g. TT[T/C]AGGG) as expected from previous analysis [22]. Cluster 2 made up 1.8% of the genome and was enriched only for *simple_repeats_C* (*p* = 1.47e-176). This relates to a previously unidentified feature of the genome: 63 polyC repeats of ~ 20 nucleotides. Twenty-eight of these repeats were found in introns, while others tended to lie close to genes. Here, GDA makes clear the alteration in genome architecture between closely related species, while also identifying previously hidden features.

### Identification of repeat-rich bands and large gene-poor subtelomeres in *Eimeria tenella*

*Eimeria spp.* parasites have been found in a wide range of vertebrates and commonly cause coccidiosis in domesticated chickens. We have previously shown that their ~ 50 Mbp genomes contain a banded pattern of regions rich in CAG and telomere-like (TTTAGGG) repeats [23]. Coding regions are enriched for the CAG repeat, which tends to encode Homopolymeric Amino Acid Repeats (HAARs) of alanine, serine or glutamine and litter even very well-conserved genes. We recently sequenced the genome of *Eimeria tenella* using long reads and Hi-C scaffolding, producing a nearly chromosomal assembly [24].

**Fig. 5:**
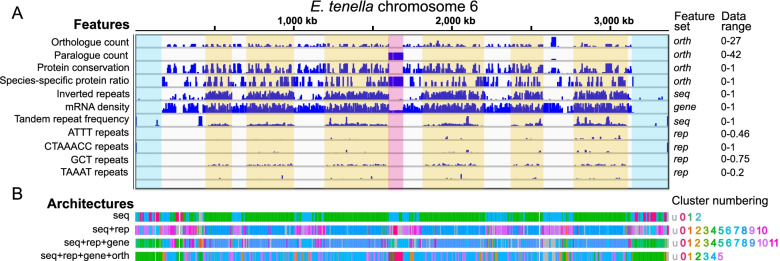
Repeat-rich bands and gene-poor subtelomeres of *Eimeria tenella* are captured more or less well by different feature sets. **A** A number of features are shown in 5kbp windows across chromosome 6 of *E. tenella*. The repeat-rich bands, defined here by GCT (CAG) repeats are highlighted in yellow. The gene-poor subtelomeres are highlighted in blue and a *sag* multigene family array in pink. Data range indicates minimum and maximum values for the y axis of each feature. **B** Four different architectures, based on different feature sets are shown below. The *seq*, *seq* + *rep* and *seq* + *rep* + *genes* feature sets capture the repeat-rich regions very well, with the last of these also capturing the gene-poor subtelomeres. The *seq* + *rep* + *gene* + *orth* feature set does not capture the repeat-rich regions in a single cluster but instead focuses more on whether a window contains more well-conserved genes or not. It retains the cluster identifying the gene-poor subtelomeres and highlights arrays of *sag* genes

We investigated whether GDA was able to identify the repeat rich bands and other distinctive features in this genome using the new chromosome-scale assembly. Using the *seq* feature set resulted in three clusters. Figure 5 shows that this simple input was sufficient to define the repeat-rich bands across the genome. 95.2% (3,927) of genes containing HAARs fell into cluster 1. This highlights that when using only simple features, GDA is able to accurately capture this aspect of genome architecture, and furthermore, that *E. tenella* genome architecture is dominated by this feature.

**Fig. 6:**
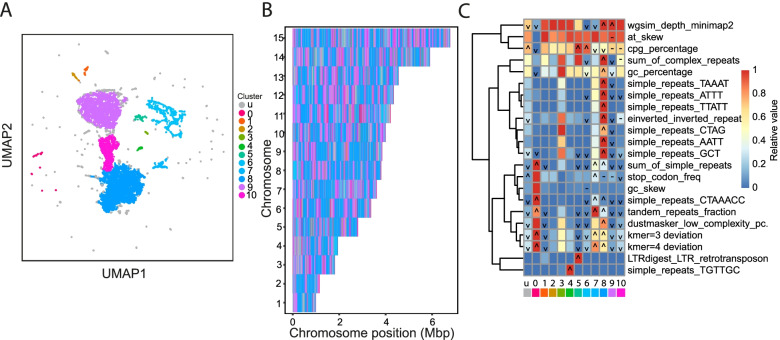
GDA analysis of *Eimeria tenella* with the *seq* + *rep* feature set. **A** Analysis of *E. tenella* with the *seq* + *rep* feature set identified 11 clusters. The majority of the genome was separated into three or four clusters found in bands across each chromosome (**B**). **C** These include the repeat rich region (cluster 8; dark blue), a cluster which is similar but lacks repeats (9; purple) and an intermediate cluster (10; magenta) which is enriched for *sum of complex repeats* and *inverted repeats*, but not the GCT/CAG and telomere-like (CTAAACC) repeats found in cluster 8. In the heatmap colours indicate the relative value of the feature in each cluster (red = highest, blue lowest), icons indicate significance (‘∧’ = KS test greater p-value <  = 1e-20, ‘∨’ = KS test lesser *p*-value <  = 1e-20, ‘-’ = great and lesser p-values <  = 1e-20)

To better understand the repeats present in the different regions, we ran GDA again, adding in the *rep* feature set (silhouette score = 0.32; Fig. 6A-C). We saw that cluster 8 (41.69% of the genome) was enriched for simple_repeats_CTAAACC (*p* = 0; i.e. the telomere-like repeat) and simple_repeats_GCT (*p* = 0; i.e. CAG repeat) as well as inverted repeats and several complex repeat families (Fig. 6C). This cluster overlapped 93.8% of HAARs (26,728/28,483). With this feature set, cluster 9 represented the gene-rich parts of the genome lacking repeats (23.61%), while cluster 10 (9.04%) —intermediate between clusters 8 and 9 in the UMAP plot — was enriched in inverted repeats and sum of complex repeats. Cluster 5 captured the LTR retrotransposons, which are not a common feature in apicomplexan genomes and were first identified in *E. tenella* and then subsequently in avian malaria parasites [25, 26]. Cluster 4 was enriched for TGTTGC repeats, which were the only enriched simple repeats to not colocalise in the repeat-rich cluster 8 regions, instead being more evenly dispersed throughout the chromosomes. On chromosome 6 it is repeated between tRNA genes in a tRNA cluster, but otherwise does not have an obvious pattern.

Adding in gene features (*seq* + *rep* + *genes*) distinguished gene-poor regions at the subtelomeres and internally within chromosomes (Fig. 5). Clusterings with high silhouette scores and relatively few unclassified windows failed to distinguish the repeat-rich regions. We picked parameters which resulted in a separate cluster for windows intermediate between repeat-rich and repeat-poor clusters, with a relatively moderate 13.68% unclassified windows and silhouette score = 0.18 (*n* = 10, c = 50; Sup Fig. 3). This allowed the identification of gene-poor subtelomeric (and sometimes internal) regions with repeat-rich regions still well-characterised (26,566/28,483 HAARs in cluster 9; Fig. 5). Gene-poor subtelomeric regions have not previously been described as a feature of *Eimeria* chromosomes. These subtelomeric gene deserts (clusters 4 and 5) have high CpG content and cluster 5 has high stop codon frequency, while cluster 4 has low uniqueness, despite not being enriched for any particular repeat families.

**Fig. 7:**
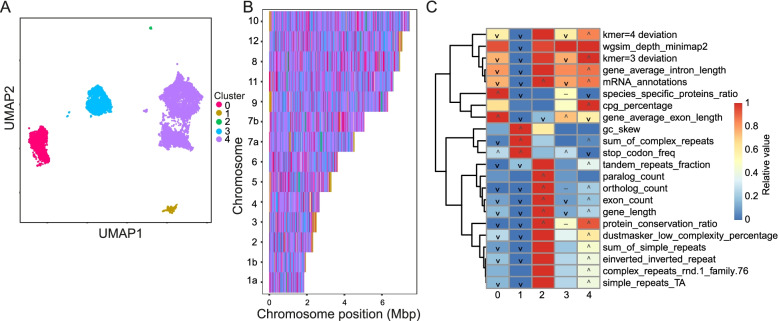
GDA analysis of *Toxoplasma gondii* highlights gene-poor subtelomeres and gene family-rich islands. **A** Using the *seq* + *rep* + *gene* + *orth* feature set, the *T. gondii* genome separated into 5 distinct clusters. **B** Cluster 1 (gold) was often found at the ends of chromosomes and was typified by low numbers of mRNA annotations, high GC skew, complex repeats and stop codon frequency (**C**). This is similar to what we see in *E. tenella* subtelomeres. In the heatmap colours indicate the relative value of the feature in each cluster (red = highest, blue lowest), icons indicate significance (‘∧’ = KS test greater *p*-value <  = 1e-20, ‘∨’ = KS test lesser *p*-value <  = 1e-20, ‘-’ = great and lesser *p*-values <  = 1e-20)

We wanted to determine whether gene poor subtelomeres were also present in other *Coccidia* and so we ran GDA on the related species *Toxoplasma gondii* with *seq* + *gene* + *rep* + *orth* feature sets*.* The genome resolved into 5 distinct clusters, with no unclassified windows (Fig. 7). Chromosomes often ended in gene-poor regions falling into cluster 1 (mRNA_annotations lower than other regions; *p* = 5.6e-310). These had high stop codon frequency (*p* = 5.35e-74), high GC skew (*p* = 8.05e-30) and were enriched for complex repeats (*p* = 1.03e-26), although no individual repeats in particular, much like *E. tenella*.

Next, we ran GDA on *E. tenella* including the *orth* feature set (*seq* + *rep* + *gene* + *orth*) to see if we could identify patterns of gene conservation amongst the complexity of the repeat regions (6 clusters, *n* = 10, c = 100, 1.9% windows unclassified). The gene-poor subtelomeres remained well classified (Fig. 5), but the *sag* gene arrays on chromosomes 6, 9 and 11 were now also well-captured by cluster 0. Of 78 *sag* genes, 51 overlapped cluster 0 windows. In this clustering the repeat-rich cluster was lost. Instead, much of each chromosome was split into windows with well-conserved genes (cluster 4 – 46.61% windows) or more species-specific genes (cluster 5 – 15.61%). Both these clusters were enriched for “simple_repeat_GCT” (i.e. CAG repeats; KS-test one-sided p-value 1.03e-234 for cluster 4, 1.43e-82 for cluster 5).

The *E. tenella* genome highlights how some important properties of genome architecture are not well captured with a single parameter set. Using different feature sets, and parameters such as window size, enabled different aspects of genome architecture to be represented.

### GDA can be run on large genomes and with high resolution

We measured the time taken to run the genomic feature extraction pipeline of GDA with the genome assemblies of four different species representing a range of genome sizes: *Plasmodium falciparum* (~ 23 Mb), *Caenorhabditis elegans* (~ 100 Mb), *Schistosoma mansoni* (~ 410 Mb) and *Homo sapiens* (~ 3300 Mb) (Table 1). In each case 5kbp windows were used, meaning that for *H. sapiens*, features were calculated over 654,762 windows. Memory requirements were roughly correlated with genome size and were not greatly affected by repeat finding. Run time was roughly correlated with genome size, however *C. elegans* took longer to process than *S. mansoni*. The major factor contributing to long run times was using RepeatModeler to identify repeats de novo* (rep* feature set*)*. Without this step, analysis of the *P. falciparum* genome was completed in 17 min and the human genome in less than 12 h. When de novo repeat finding was included these analyses took ~ 11 h and 87 h respectively. However, it is clear that GDA can be run effectively on large genomes with resources commonly available on bioinformatics compute clusters, even including time-intensive repeat finding. In an analysis of the human genome with 50kbp windows, GDA clearly identified centromeres and pericentromeric repeat-rich regions known to be important features of chromosome architecture [27] (Sup Fig. 4). The bulk of the genome was divided into regions rich in protein coding genes and those lacking protein coding genes. These clusters highlighted chromosome 19 as particularly dense with protein coding genes and chromosome 13 as having a relative deficit of protein coding genes. A fifth cluster highlighted regions primarily containing retrotransposons, an especially common feature of the human genome [28].

**Table 1:** Resource requirements for running the GDA feature extraction pipeline on a range of genomes. The GDA feature extraction pipeline was run with four genomes of different sizes. De novo repeat detection had a large effect on run time while genome size caused increases in both run time and memory usage

Feature set		***P. falciparum***	***C. elegans***	***S. mansoni***	***H. sapiens***
	Assembly size (Mbp)	23.33	100.29	409.57	3272.09
*Seq* + *gene* + *orth* (without RepeatModeler)	Run time	17 min	1 h 1 min	1 h 37 min	11 h 42 min
	CPU time (s)	2475.22	13,444.56	16,204	126,110
	Max memory use (Mb)	4573	8878	11,738	145,277
*Seq* + *gene* + *rep* + *orth* (with RepeatModeler)	Run time	11 h 16 min	8 h 59 min	41 h 13 min	86 h 6 min
	CPU time (s)	408,912	238,074	1,184,172	1,862,049.88
	Max memory use (Mb)	4278	9326	11,730	128,683

## Discussion

We have presented a new tool, GDA, which decomposes a genome sequence into windows, identifying those with similar properties and enabling the characterisation of genomic architectural features. This is achieved most simply using properties derived from the genome sequence alone, but a wide range of additional properties can be used as input. We have shown that GDA recapitulates the well-described architecture of the malaria parasite *Plasmodium falciparum* and in doing so defines regions of interest that can be further explored. The description of the *P. falciparum* genome was robust to different feature sets, suggesting that each part of the genome has multiple features distinguishing it from other regions which are correlated with each other. In the *Eimeria tenella* genome, GDA analysis highlighted the banded pattern of repeats observed previously [23, 25] and shows for the first time that it is present across all chromosomes. A previous attempt to define these regions involved arbitrary cutoffs, but GDA provides a straightforward and data-driven approach to define the repeat-rich regions. This will facilitate the comparison of different *Eimeria* spp. genomes in studying the evolution of these repeat-rich regions across species.

The power of GDA lies in the way it allows visualisation of genome architecture to suggest hypotheses about genome function and evolution. Applied to closely related species, substantial changes in organisation of genomic features can be quickly recognised (as in the example of *P. vivax* and *P. knowlesi*). The drivers of these features can be readily determined and investigated (as in the CAG repeats in protein-coding genes of *E. tenella*). This makes GDA a powerful tool for any de novo genome sequencing or comparative genomics project involving well-assembled genomes. We foresee a range of applications such as sex and accessory chromosome identification, genome assembly curation and interpretation of epigenomic datasets (e.g. ChIP-seq/ATAC-seq). In fact, similar approaches to ours have been used to analyse patterns of chromatin modifications in isolated genomic regions [29] and patterns of relatedness across genomes [30]. However, we are not aware that similar approaches have been applied to characterise genome-wide architecture and we have not found any tool which has this aim.

When considering application of GDA for different purposes and on different sizes of genome, window size is an important parameter. The choice of window size should reflect the resolution of features that the user is interested in. A window size of 1kbp in a 100Mbp genome may reflect individual parts of genes such as separate exons, introns and promoters which would be appropriate for understanding patterns in many types of ChIP-seq data. On the other hand, windows of 5-10kbp may reflect one or a handful of genes or complex repeats per window, while 1Mbp windows will reflect more broad aspects of genome architecture.

All Apicomplexan genomes appear to be relatively small and compact, however their architectures are diverse. Unlike some larger genomes, in which there is little linear architectural coherence based on sequence properties, repeats and homology, these genomes display quite definite ordering. Current work on mammalian genomes suggests that important aspects of architecture relating to the control of gene expression are manifest in the third dimension, i.e. the arrangement of the linear chromosomes in space [31]. These arrangements can be assayed by techniques such as Chromatin Conformation Capture (e.g. Hi-C). Although not linear in nature, the data from these assays could be reframed as linear features (for instance regions of high connectivity between chromosomes) and used as input to GDA. GDA is not limited to the examination of apicomplexan genomes, or even just eukaryotes, but can be used with any DNA sequence. Despite the large amount of computation involved, GDA can be run on large genomes with large feature sets in about a week. The most time-consuming step is repeat finding, and we are exploring alternatives that would bring the overall run time down substantially. Despite its large size and great degree of complexity, GDA is able to identify the major features of human genome architecture.

## Methods

### Genome decomposition analysis pipeline

Version 1.0 of GDA was used throughout, with default parameters unless otherwise specified. A window size of 5kbp was used throughout as this represents roughly the size of a gene in apicomplexans (e.g. Plasmodium spp.). The GDA v1.0 code was cloned from a private git repository to a Linux server and a Conda environment that includes all software dependencies established using the *create_gda_conda_env.py* script provided. This installation was used for running the feature extraction, clustering and analysis parts of the pipeline.

The pipeline extracts the values of various sequence features (e.g. GC content) with a sliding window (default size 5kbp) along all sequences in the assembly. The values are stored as separate bedgraph files (one per feature). The pipeline consists of a master script that is written in Nextflow [32]. The rest of the code of the pipeline has been written mostly in Python. The Nextflow script triggers multiple third party software tools that are used to detect genomic features. As an alternative to using the Conda environment, the pipeline and its dependencies are packaged as a Singularity [33] image, thus simplifying its installation in a shared environment.

Using a genome assembly FASTA file as the input, the genomic feature extraction pipeline determines low complexity sequence content using Dustmasker 1.0.0 [34], tandem repeat content using Tandem Repeats Finder 4.09.1 [35], 10 × coverage of simulated reads using WGSIM 1.0 (https://github.com/lh3/wgsim), retrotransposons using LTRharvest and LTRdigest from GenomeTools 1.6.1 [36], inverted repeats using einverted from EMBOSS 6.6.0 [37] and repeat families using either RepeatMasker + RepeatModeler 2.0.1 [38] or Red (05/22/2015) + MeShClust2 2.3.0 [39, 40]. GC%, AT skew, GC skew, and the frequency of CpG dinucleotides, stop codons and telomeric motifs in each window are determined using Python code. If the user does not provide the pipeline with a gene annotation file, the pipeline can annotate genes itself using Augustus 3.3.3 [41], tRNAscan-SE 2.0.6 [42], and Barrnap 0.9 [https://github.com/tseemann/barrnap]. It is possible to provide hints for Augustus using annotation transfer from a GFF3 file of a related genome with Liftoff 1.6.1 [43]. With additional input data, the pipeline can detect ectopic mitochondrial and apicoplast sequences using BLAST 2.10.1 [34], and RNA-Seq read coverage using HISAT2 2.2.1 [44]. If the user provides proteome FASTA files of species that are related to the target species, the pipeline can run OrthoMCL 1.4 [45]. A more detailed description of the variables can be found in Supplementary Table 1. Note that telomeric motifs, stop codons and kmers are not counted if they are broken up by a border between two windows. However, in the OrthoMCL results analysis part (when calculating the values of variables per gene in the window) a gene that is split between two windows is counted as a part of both windows.

The code for the dimensionality reduction and clustering of the data from genomic windows uses the Python UMAP [17] and HDBSCAN [18] libraries. The scaling of variables before running UMAP is done using MinMaxScaler from the scikit-learn package [46].

In the script for optimising the clustering parameters (gda_parameters.py), Silhouette score, Davies-Bouldin index and Calinski-Harabasz score are calculated for each clustering result using scikit-learn. These scores help to find the clustering settings that work the best for separating the genomic windows into distinct clusters.

After determining the optimal settings for n_neighbors and minimal cluster size, the pipeline runs the final clustering. Kolmogorov–Smirnov test is used to determine whether the distribution of values of a variable in a GDA cluster is significantly different from the distribution of the values of the same variable in the rest of the genomic windows. The test is performed using the ks_2samp function from the scipy package [47]. The Fisher test with Benjamini–Hochberg multiple hypothesis testing correction (using scipy.stats [47] and statsmodels.stats.multitest libraries [48] are used to determine if some types of cluster junctions occur with a different frequency than what is expected by chance. For example, this test yields a statistically significant result when windows belonging to a given cluster are located next to windows belonging to the same other cluster significantly more often than expected by chance.

While the clustering and visualisation parts of the GDA pipeline rely on bedgraph files, none of the third party software tools used by GDA produce output files in bedgraph format. We therefore use Python code written for the GDA pipeline to derive bedgraph files from the diverse set of output files produced by the third party tools. In some cases, the output of a software tool is first converted to GFF format and then the GFF file is converted to a bedgraph file. All bedgraph files corresponding to one assembly are merged into a tab-separated table. The code for merging bedgraph files into a table and for downsampling the table has been written in C +  + instead of Python, in order to gain execution speed.

In this work, we distinguish four different feature sets: *seq* requires only the genome sequence as input, *gene* features are derived from a set of gene annotations (e.g. mRNA, rRNA, tRNA etc. features in a GFF file), *rep* features derived from running the RepeatModeler repeat classification and analysis tool, *orth* derived from running the OrthoMCL tool for determining orthologous and paralogous relationships between protein-coding genes. These feature sets are frequently combined, as stated. In this work “full feature set” refers to the combination of these four feature sets, e.g. *seq* + *gene* + *rep* + *orth*. GDA is capable of generating additional feature sets and any arbitrary genome data tracks can be added to incorporate novel features.

### Datasets

Genome sequences and annotation for the following species were downloaded from VEuPathDB release 51 (https://toxodb.org/toxo/app/downloads/release-51/)—*Plasmodium falciparum* 3D7, *P. knowlesi* H, *P. chabaudi* AS, *P. vivax* P01, *Toxoplasma gondii* ME49, *Babesia bovis* T2Bo, *B. microti* RI, *Theileria annulata* Ankara, *T. parva* Muguga and *Cryptosporidium parvum* Iowa II. Features in the GFF files labelled *protein_coding_gene* were changed to *gene*. *Eimeria tenella* Houghton data was downloaded from ENA (https://www.ebi.ac.uk/ena/browser/view/GCA_905310635.1). For OrthoMCL runs (excluding large genome analysis), all the above species were included.

### Analysis of *Plasmodium falciparum*

The feature extraction module of GDA was initially run using just the sequence as input, producing the following features: at_skew, cag_freq, cpg_percentage, dustmasker_low_complexity_percentage, einverted_inverted_repeat, N_percentage, gc_percentage, gc_skew, kmer_deviation_kmer_size_3, kmer_deviation_kmer_size_4, LTRdigest_protein_match, LTRdigest_LTR_retrotransposon, stop_codon_freq, tandem_repeats_fraction, telomere_freq, wgsim_depth_minimap2. A description of these features is available in Supp. Table 1.

The clustering_params function of GDA was used to determine suitable clustering parameters, with all combinations of *n neighbours* (*n*) = {5, 10, 15, 20} and *minimum cluster size* (c) = {50, 100, 200 500} explored. Parameter values were chosen to minimise the percentage of unclassified windows and maximise the silhouette score. This was achieved with *n* = 5 and c = 50. Feature extraction was also performed with the addition of gene annotations (*seq* + *gene*), resulting in the following additional features: exon_count, gene_average_exon_length, gene_average_intron_length, gene_length, mRNA_annotations, pseudogene_annotations, rRNA_annotations and tRNA_annotations. Clustering parameters were *n* = 10 and c = 40. To this feature set, repeat identification with RepeatModeler was added (*seq* + *gene* + *rep*), incorporating sum_of_simple_repeats, sum_of_complex_repeats, as well as numerous, specific simple and complex repeat family features. Clustering parameters for this feature set were *n* = 15, c = 50. The final feature set added features derived from an analysis of orthologues across the Apicomplexan phylum: apicomplexa_ortholog_count, apicomplexa_paralog_count, apicomplexa_protein_conservation_ratio and apicomplexa_species_specific_proteins_ratio (*seq* + *gene* + *rep* + *orth*). Here, the clustering parameters were chosen as *n* = 20, c = 50.

We wanted to determine whether cluster 3 (var/rif genes) and 4 (smaller multigene families) regions in the seq + gene + rep + orth run of *P. falciparum* were more or less well covered by HP1 chromatin modifications in internal regions versus subtelomeres. We defined subtelomeric windows as those within 200kbp of chromosome ends. To test whether there was a difference in HP1 occupancy between subtelomeric and internal multigene family regions, bedgraph files of log2 ratios of HP1 in trophozoites were downloaded from PlasmoDB, originally derived from [49]. We used *bedtools intersect* to identify genes overlapping windows of each cluster. Boxplots were drawn using the *graphics* v4.0.2 package in R. Kolmogorov–Smirnov tests, using the *stats* v4.0.2 package in R, were used to determine statistical significance.

### Analysis of *P. vivax* and *P. knowlesi*

Full feature sets (*seq* + *gene* + *rep* + *orth*) were used for *P. vivax* and *P. knowlesi*. For *P. vivax* we chose parameters *n* = 20, c = 50, for *P. knowlesi* *n* = 10, c = 50. Clustering parameters were chosen using the clustering_params function of GDA as for other species.

### Analysis of *Eimeria tenella*

We used parameters *n* = 10 and c = 100 with the *seq* feature set, resulting in exclusion of 2.74% of windows and a silhouette score of 0.53. The default CAG repeat feature was excluded because this feature was originally added specifically to help identify repeats in *Eimeria* spp. Here, we wanted to demonstrate that these repetitive regions could be identified without prior knowledge. We added *rep* features (*n* = 5, c = 50, silhouette score = 0.32), then *gene* features (13.68% unclassified windows and silhouette score = 0.18, *n* = 10, c = 50), then *orth* features (6 clusters, *n* = 10, c = 100, 1.9% windows unclassified).

Homopolymeric Amino Acid Repeats (HAARs) were identified using Python regular expressions, looking for runs of A, S, Q, L and N of at least 7 in predicted protein sequences. There were 13,389 A, 9,404 Q, 5,350 S, 334 L and 6 N repeats.

### Analysis of *Toxoplasma gondii*

Non-chromosomal contigs were removed from the assembly. The *seq* + *gene* + *rep* + *orth* feature set was used with parameters *n* = 20, c = 50, resulting in 5 clusters, with no unannotated windows.

### Analysis of large genomes

The GDA feature extraction pipeline was run with four genomes of increasing size, with and without RepeatModeler (*rep* feature set) to show how resource requirements scale. Each was run with orthologue analysis (*orth*), genome annotation (*gene*) feature sets as well as NUclear Mitochondrial DNA (NUMT) identification. All jobs were executed on the Wellcome Sanger Institute compute farm with Intel(R) Xeon(R) Gold 6226R CPU @ 2.90 GHz processors.

and up to 16 threads. Genomic windows size was 5 kbp in all runs—which represents 654,762 windows for *H. sapiens*. Gene annotations were read from existing GFF files from the same origin as the assembly FASTA files (PlasmoDB, NCBI or WormBase ParaSite).

*Plasmodium falciparum* 3D7 (PlasmoDB release 43) was used with the Pf_M76611 (PlasmoDB) mitochondrial genome reference and reference proteomes *P. chabaudi chabaudi* AS, *P. ovale curtisi* GH01, *P. gallinaceum* 8A, *P. malariae* UG01, *P. berghei* ANKA, *P. vivax* P01 (from PlasmoDB release 52). *Caenorhabditis elegans* (RefSeq GCF_000002985.6) was used with mitochondrial sequence NC_001328.1 (NCBI) and predicted proteomes GCF_000001215.4 Release 6 (*Drosophila melanogaster*), GCF_000146045.2 R64 (*Saccharomyces cerevisiae*) and GCF_000001405.39 GRCh38.p13 (*Homo sapiens*) from NCBI, GCA_900184235.1 (*Oscheius tipulae*) and GCA_000469685.2 (*Haemonchus contortus*) from GenBank and PRJEA36577.WBPS14 (*Schistosoma mansoni*) from WormBase ParaSite. *Schistosoma mansoni* (WormBase ParaSite release 14, assembly Smansoni_v7) was used with mitochondrial sequence NC_002545.1 (NCBI) and predicted proteomes PRJDA72781.WBPS14 (*Clonorchis sinensis*), PRJEB527.WBPS14 (*Schistocephalus solidus*), PRJEB122.WBPS14 (*Echinococcus multilocularis*), PRJEA34885.WBPS14 (*Schistosoma japonicum*), PRJNA179522.WBPS14 (*Fasciola hepatica*), PRJEB124.WBP from WormBase ParaSite [50]. Homo sapiens (NC_012920.1; NCBI) was run with mitochondrial sequence NC_012920.1 (NCBI) and predicted proteomes GCF_000002035.6_GRCz11 (*Danio rerio*), GCF_001663975.1 (*Xenopus laevis* v2), GCF_000001635.27_GRCm39 (*Mus musculus*) from NCBI.

For clustering analysis of the human genome, features were calculated in 5kbp windows and then merged into 50kbp windows. Tracks for individual repeat families were excluded but *sum of simple repeats* and *sum of complex repeats* features were used. Suitable clustering parameters were chosen using the *clustering_params* tool, with n_neigbors = 50 and minimum cluster size = 500.

## Supplementary Information

**Additional file 1: Supplementary Figure 1.** Effect of varying *N neighbours* (n) and *minimumcluster size* (c) parameters on clustering of 5kb windows from *Plasmodium falciparum* with the *seq* feature set. Values for thepercentage of unclassified windows (U) and the silhouette score (S) are shownbeneath each UMAP plot. We aimed in this work to identify clustering parameterswhich resulted in a small percentage of unclassified windows, a high silhouettescore and a reasonable number of clusters. Here we picked *n* = 5, c = 50, wherethere were no unclassified windows and the silhouette score was reasonablyhigh. Other clusterings had higher silhouette scores (e.g. *n* = 20, c = 50), buthad fewer clusters, suggesting they might be missing an interesting architecturalfeature captured by the *n* = 5, c = 50 clustering. **Supplementary Figure 2.** Heterochomatin Protein 1 occupancy inclusters 3 and 4 of *P. falciparum seq+gene+rep+orth* analysis. (**A**)HP1 occupancy is generally high in cluster 3 windows, but slightly lower insubtelomeric than internal locations (Kolmogorv-Smirnov test, D- 0.29348, p =0.007814). (**B**) HP1 occupancy is generally lower in cluster 4 windows comparedto cluster 3. Subtelomeric cluster 4 windows tend to have higher HP1 occupancythen internal ones. **Supplementary Figure3.** Effect of varying *N neighbours*(n) and *minimum cluster size* (c)parameters on clustering of 5kb windows from *E. tenella* with the *seq+rep+gene*feature set. A range of *n* and *c* parameters were evaluated to determinea good clustering of genomic windows. U = unclassified window percentage, S =silhouette score. Selecting n = 10 and c = 50 allowed the identification ofgene-poor subtelomeric (and sometimes internal) regions with repeat-richregions still well-characterised. **Supplementary Figure 4.** GDA analysis of the human genome with 50kbwindows. (**A**) A UMAP plot of all 50kb windows of the human genome showsthat it separates into five distinct clusters. (**B**) Key features such ascentromeres (cluster 0 in red) and pericentromeric segmental duplications(cluster 3 in blue) are captured. (**C**) A heatmap of features associated witheach cluster shows that the centromeric cluster (0) is enriched for complexrepeats and skewed nucleotide content (high gc_skew and kmer deviation). Thesegmentally duplicated regions (cluster 3) are indicated by high numbers ofpseudogenes and inverted repeats and high GC content. **Supplementary Table 1.** Variables extractedby the genomic feature extraction pipeline of GDA. Each feature that can be generated by the GDAfeature extraction pipeline is described here, highlighting whether it isincluded in a particular feature set.

## Data Availability

The datasets generated and analysed during the current study are available in the GiHub repository https://github.com/eeaunin/gda. Project name: GDA (Genome Decomposition Analysis). Project home page: https://github.com/eeaunin/gda Archived version: https://github.com/eeaunin/gda/releases/tag/v1.0 Operating system(s): Genomic feature extraction pipeline: Linux. The Shiny web app: Linux and MacOS. Programming language: Python, Nextflow, R, C +  + Other requirements: Dependencies for the genomic feature extraction pipeline are the following. If installing using Conda (versions with which it has been tested in are brackets): Conda (4.10.3), Python3 (3.7.10), git (2.17.1), g +  + (4.9.1 or later). If using the Singularity image (versions with which it has been tested in are brackets): Singularity (3.6.4), Nextflow (0.30.1). Dependencies for the Shiny web app: Conda (tested with 4.10.3) and the following R packages from Conda repositories. r-shiny = 1.5.0 r-ggplot2 = 3.2.1 r-gplots = 3.0.3 r-rjson = 0.2.20 r-reshape2 = 1.4.3 r-gridextra = 2.3 r-scales = 1.0.0 r-svglite = 1.2.3 License: MIT License. Any restrictions to use by non-academics: None.
